# Is there an alternative to the indirect immunofluorescence ANA HEp-2 assay for the diagnosis of connective tissue diseases?

**DOI:** 10.3389/fimmu.2025.1669124

**Published:** 2025-11-03

**Authors:** Tamazouzt Hadjout, Lydia Lamara Mahammed, Meriem Saad, Amel Zemmour, Nadia Tamechmacht, Ghalya Bousbia, Halima Ismail, Nawel Dahmani, Ines Allam, Reda Djidjik

**Affiliations:** ^1^ Department of Medical Immunology, Beni Messous University Hospital Center, Algiers, Algeria; ^2^ Faculty of Pharmacy, the University of Health Sciences, Algiers, Algeria

**Keywords:** anti-nuclear antibodies, ANA-associated rheumatic diseases, chemiluminescence, IIF, solid phase immunoassays

## Abstract

**Background:**

Anti-nuclear antibodies (ANA) are serological hallmarks in the diagnosis of ANA-associated rheumatic diseases (AARD). For many years, indirect immunofluorescence (IIF) on HEp-2 cell substrate has been regarded as the gold standard method for ANA detection. Nowadays, several solid phase immunoassays (SPA) have been developed for ANA screening. The aim of this study was to evaluate three automated assays as potential alternatives to the IIF HEp-2 assay for ANA detection in the diagnosis of AARD.

**Patients and methods:**

This study included 271 patients referred to our department for routine ANA testing: 94 patients with confirmed AARD, 144 in whom AARD was excluded and 33 with an uncertain AARD diagnosis. For all sera, ANA detection was initially performed using an IIF HEp-2 assay (EUROIMMUN^®^, Lubeck, Germany), then assessed by two chemiluminescence immunoassays (CLIAs) on the MAGLUMI^®^ X3 (Snibe, Shenzhen, China) and the iFlash 1800^®^ (YHLO, Shenzhen, China), and an automated enzyme immunoassay (EIA) UNI^®^ (NeoMedica, Nis, Serbia). For identification, we performed anti-ENA and anti-DNA assays using the CLIA MAGLUMI^®^ X3 or the ELISA EUROIMMUN^®^ assay.

**Results:**

The highest positivity rate was found with the MAGLUMI in the AARD group, with the highest concordance rate with IIF (77.9% *vs*. 73.4% with UNI, and 71.2% with iFlash). The three automated ANA assays showed weak agreement with the IIF assay (0.454 ≤ ĸ ≤ 0.551). The three ANA assays showed excellent performance in discriminating between AARD and non-AARD cases (AUC>0.9 for each system). At the manufacturer’s cut-off values, the MAGLUMI assay showed the highest sensitivity (95.7%), and the highest specificity was found with the iFlash (94.4%). Only the MAGLUMI assay showed a negative likelihood ratio <0.1, whereas the UNI and the iFlash ANA assays showed a high positive likelihood ratio (≥10).

**Conclusion:**

These findings suggest that SPA can serve as a complementary approach to IIF for ANA screening in the diagnosis of AARD. The MAGLUMI assay could be used for initial screening alongside IIF, depending on the clinical context. A proper adjustment of the threshold of the MAGLUMI ANA Screen assay may improve its specificity and limit false positive results.

## 1 Introduction

In immunology laboratories, the diagnostic algorithm for connective tissue diseases typically begins with screening for antinuclear antibodies (ANA). Several societies, including the American College of Rheumatology (ACR), the European Autoimmunity Standardization Initiative (EASI), the International Union of Immunological Societies (IUIS), the World Health Organization (WHO), the Arthritis Foundation and the Centers for Disease Control and Prevention (CDC), recognize indirect immunofluorescence (IIF) on Human Epithelial cells type 2 (HEp-2) as the gold standard for ANA detection (1, 2).

Despite its widespread use, the IIF assay has notable limitations. The IIF assay is a time-consuming and laborious technique. It has a high inter-observer variability and subjective pattern interpretation. To address this, efforts have been made to harmonize the HEp-2 pattern nomenclature (3) and to develop automated microscopes with intelligent digital imaging to standardize IIF reading (4, 5). Besides, IIF lacks specificity, as ANAs can be detected in patients with nonrheumatic diseases and can also be present in healthy individuals (6).

Nowadays, more and more solid phase assays (SPA) for the screening of ANA have emerged. The majority of them have the advantage of being automated, allowing time savings and objectivity in the interpretation of results. Moreover, SPA adopt different technical principles, including chemiluminescence immunoassays (CLIA), enzyme immunoassay (EIA), and multiplex tests. These assays utilize diverse antigenic substrates—such as purified antigens, recombinant antigens, and cell extracts—to enhance sensitivity, a critical factor for screening assays (6–8). Ultimately, SPA are specifically designed to detect autoantibodies associated with ANA-related rheumatic diseases (AARD), thereby improving their diagnostic specificity (6).

Standardization of these emerging assays is highly recommended (6, 9). In this perspective, we aimed to evaluate three automated assays: two based on CLIA and one based on EIA for the detection of ANA by comparing them to the benchmark assay (IIF). Furthermore, the positivity of each assay was confirmed by completing the identification panel and we analyzed the discrepant results by comparing them with the clinical data and the final diagnosis.

## 2 Materials and methods

### 2.1 Study population

The study population consisted of 271 samples of patients referred to the Immunology Department of Issaad Hassani University Hospital in Algiers for ANA testing during the period between November 2023 and April 2024. Patients were referred from Internal Medicine, Rheumatology, Pneumology and Nephrology departments of the Hospital for follow up or suspicion of AARD and from outpatient consultations for routine medical checkups. Next, the subject’s medical records were retrospectively evaluated by physicians for AARD, resulting in the individualization of 3 groups (**Table 1**):

**Table 1:** Demographical and clinical characteristics of the study population (N = 271).

Characteristics	n (n/N%)
Age, mean ± SD (years) [range]	44.5 ± 16.5 [1-85]
- Children (<18 years)	- 26 (9.6)
- Adults (≥18 years)	- 245 (90.4)
Gender, n (%)	
- Males	- 43 (15.9)
- Females	- 228 (84.1)
Initial diagnosis, n (%)	
- AARD	- 94 (34.7)
• SLE	• 31 (33)
• SjS	• 29 (30.9)
• SSc	• 19 (20.2)
• MCTD	• 8 (8.5)
• PM (AS)	• 2 (2.1)
• Association of two diseases	• 5 (5.3)
- Non-AARD	- 144 (53.1)
- Undetermined	- 33 (12.2)

AARD, antinuclear antibody-associated rheumatic disease; AS, antisynthetase syndrome; MCTD, mixed connective tissue disease; PM, polymyositis; SjS, primary Sjogren’s syndrome; SLE, systemic lupus erythematosus; SSc, systemic sclerosis.

- Group 1: 94 patients with an established diagnosis of AARD [31 with Systemic Lupus Erythematosus (SLE), 29 with Sjögren’s syndrome (SS), 19 with Systemic Sclerosis (SSc), 8 with mixed connective tissue disease (MCTD), 2 with Polymyositis (PM) and 5 patients who presented an association between 2 diseases] according to respective classification or diagnostic criteria.
- Group 2: a disease control group consisting of 144 patients for whom ANA screening was requested but in whom AARD afterwards was excluded.
- Group 3: 33 patients in whom the diagnosis was still undetermined at the time of the study.

Thirty-six percent of patients were referred from Internal Medicine (43% with AARD), 24% from Rheumatology (26% with AARD), 21% from Pneumology (16% with AARD) and 7% from Nephrology (30% with AARD) departments.

Samples were stored at -20 °C until analysis.

The study protocol was approved by the ethics committee of Issaad Hassani University Hospital.

### 2.2 Methods

#### 2.2.1 IIF assay

All samples were tested for the presence of ANA by IIF using the HEp-2 cells substrate (EUROIMMUN, Lübeck, Germany). A trained immunologist interpreted the IIF results using an immunofluorescence microscope. Positivity of the samples was based on the fluorescence intensity at a serum dilution of 1:100, as recommended by the manufacturer. IIF results were reported by specifying the titer and the pattern, in accordance with the recommendations (1).

**Figure 1:**
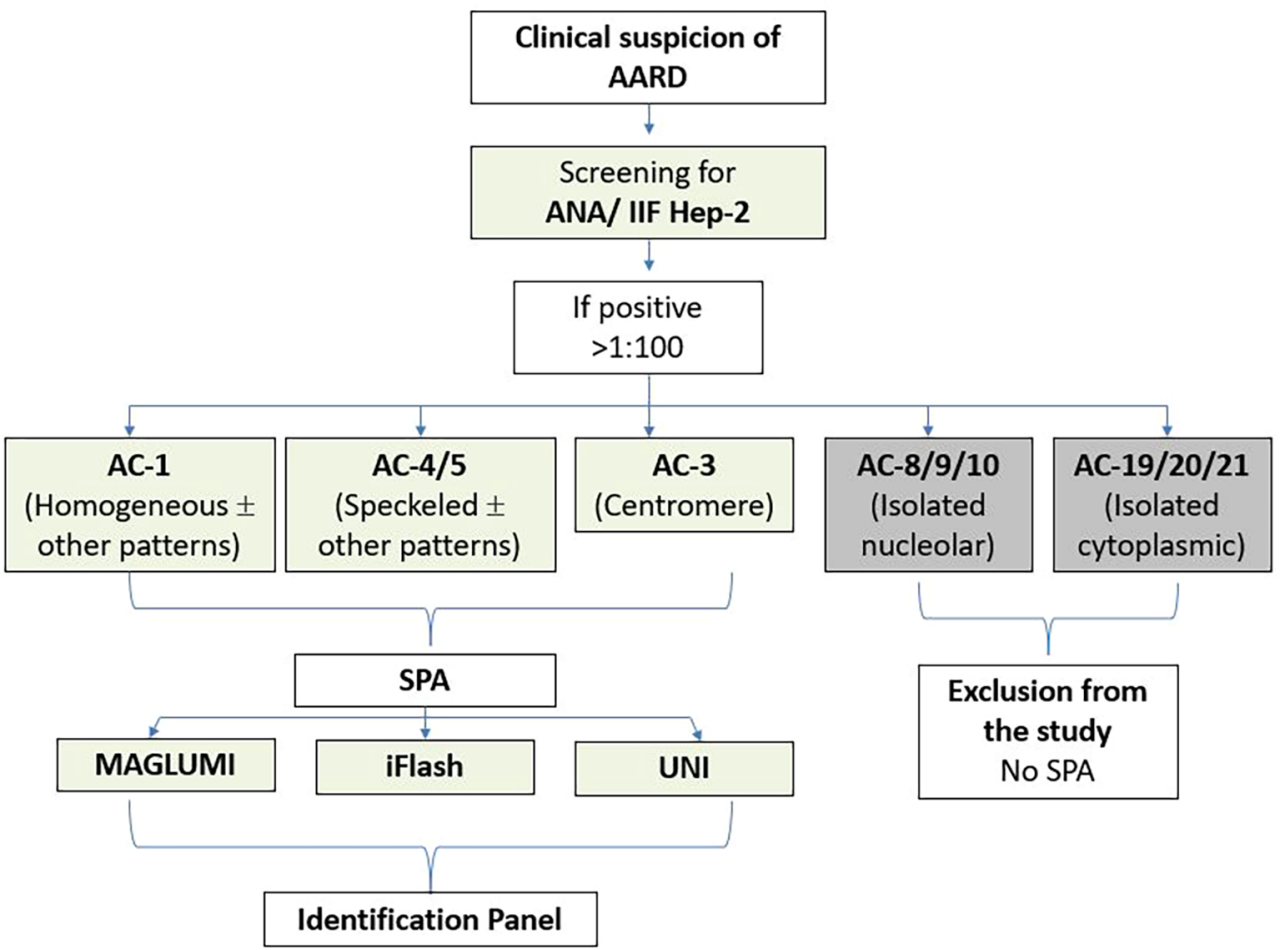
Diagram of the study protocol. AARD, antinuclear antibody-associated rheumatic disease; IIF, indirect immunofluorescence; SPA, solid phase assays. ANA, Anti-nuclear antibodies; HEp-2, Human Epithelial cells type 2.

For further analyses, we deliberately excluded sera with isolated AC-8/9/10 (nucleolar) or isolated AC-19/20/21 (cytoplasmic) patterns, since their corresponding antigenic targets are not included in the SPA reagents evaluated (e.g., RNA pol III, Ku, Mi-2, TIF1G, Fibrillarin, PM-Scl75/100, Th/To, NOR90, SRP, PL-7, PL-12, EJ, OJ, ribosomes) (**Figure 1**).

#### 2.2.2 ANA screening by MAGLUMI^®^ X3, Snibe

The MAGLUMI^®^ ANA Screen assay (Snibe, Shenzhen, China) is an *in vitro* fully-automated chemiluminescence immunoassay for the quantitative determination of IgG class antinuclear antibodies (ANA). The optimal cut-off ratio for positivity proposed by the manufacturer is ≥ 40 AU/mL. Microbeads are coated with nuclear antigens: purified dsDNA, Histones, Rib-P, nRNP/Sm, Sm, SSA, SSB, Scl70, Jo-1, Centromeres, M2-3E, together with HEp-2 cell nuclear extract.

#### 2.2.3 ANA screening by iFlash^®^ 1800, YHLO

The iFlash-ANA assay (YHLO, Shenzhen, China) is a paramagnetic particle chemiluminescent immunoassay for the quantitative detection of IgG class autoantibodies against SS-A60, SS-A52, SS-B, RNP/Sm, Scl70, Jo-1, CENP-B, Histones, dsDNA, AMA-M2. The cut-off value for positivity proposed by the manufacturer is 48 AU/mL.

#### 2.2.4 ANA HEp-2 screening by UNI®, NeoMedica

ANA-HEp-2 (NeoMedica, Niš, Serbia) is a solid phase enzyme immunoassay for the quantitative detection of IgG antibodies against HEp-2 cells. The test collectively detects anti-nuclear antibodies against a mixture of native and recombinant antigens from lysed HEp-2 cells, including dsDNA, histones, SS-A (Ro), SS-B (La), Sm, snRNP/Sm, Scl70, PM-Scl, Jo-1 and centromeric antigens. The optimal cut-off proposed by the manufacturer is 18 U/mL.

The comparative antigenic composition of the three different assays is summarized in **Table 2**.

**Table 2:** Characteristics of solid-phase assays (SPA).

Platform	MAGLUMI	iFlash	UNI
Principal of assay	Chemiluminescent immunoassay	Chemiluminescent immunoassay	Solid phase enzyme immunoassay
Cut-off value	≥ 40 UA/mL	> 48 UA/mL	> 18 A/mL
Antigenic Composition	Nuclear antigens: purified dsDNA, Histones, SSA, SSB, Sm, nRNP/Sm, Scl70, Jo-1, Centromeres, M2-3E, Ribosome-P. HEp-2 cell nuclear extract.	dsDNA, Histones, SSA60, SSA52, SSB, RNP/Sm, Scl70, Jo-1, CENP-B, RNP/Sm, AMA-M2.	Recombinant dsDNA, Histones, SSA(Ro), SSB, Sm, snRNP/Sm, Scl70, PM-Scl, Jo-1, Centromeric antigens. Lysed HEp-2 cells.

For identification, we performed follow-up testing to identify the specific autoantibodies present. We screened for anti-Extractible Nuclear antigens (ENA), including: SSA, SSB, Sm, Sm/RNP, Jo-1 and for anti-DNA using the chemiluminescence immunoassay MAGLUMI^®^ X3 (Snibe, Shenzhen, China) or the ELISA assay (EUROIMMUN, Lübeck, Germany) in samples with discrepant automated immunoassays and IIF results.

### 2.3 Statistics

Qualitative agreement between the IIF assay and each of the three SPA was calculated using Cohen’s kappa (κ) with 95% confidence intervals (CIs), which were interpreted as follows: ≤0.20, none; 0.21–0.39, minimal; 0.40–0.59, weak; 0.60–0.79, moderate; 0.80–0.90, strong; and >0.90, nearly perfect. The Spearman rank test was used to determine the correlation coefficient between the results of the different immunometric SPA evaluated (≤0.10, negligible; 0.11–0.39, weak; 0.40–0.69, moderate; 0.70–0.89, strong; and >0.90, very strong).

Receiver operating characteristic (ROC) curve analysis was performed. Sensitivity, specificity, likelihood ratios (LRs) and the optimal cut-off value were calculated. Optimal cut-off values for CLIA and automated EIA assays were obtained using the Youden index to optimize combined sensitivity and specificity.

Statistical analysis was performed using SPSS software (IBM Statistic 20.0). *p* values <0.05 indicated statistical significance.

## 3 Results

### 3.1 Prevalence of anti-nuclear antibodies

**Figure 2:**
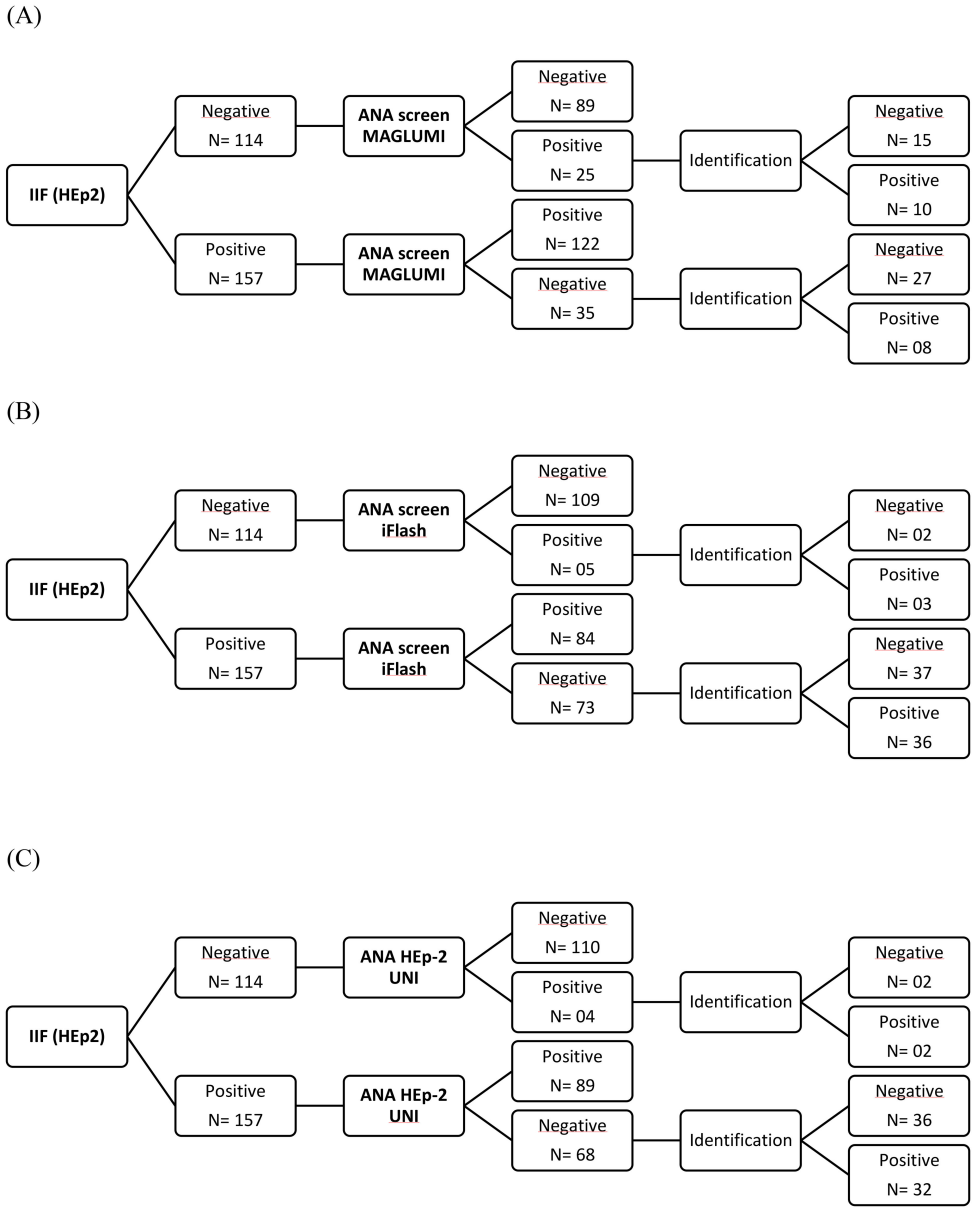
Flow charts showing the assay results among patients of the study population. **(A)** CLIA MAGLUMI results. **(B)** CLIA iFlash results. **(C)** EIA UNI results.

Three assays for ANA screening [CLIA MAGLUMI, CLIA iFlash, automated EIA UNI] were evaluated in comparison with an IIF EUROIMMUN assay in 271 patients. Results of each assay compared to the reference test (IIF EUROIMMUN) are represented in **Figure 2**. An overview of the evaluated assays results compared to the IIF assay, according to disease groups, is shown in **Table 3**.

**Table 3:** Overview of the assay results among patients of the study population.

Methods		Hep 2	
		(+)	(–)
MAGLUMI	**(+)**	122	25
		- 22 UD	- 04 UD
		- 10 non-AARD	- 21 non-AARD
		- 90 AARD	
	**(–)**	35	89
		- 07 UD	- 89 non-AARD
		- 24 non-AARD	
		- 04 AARD	
iFlash	**(+)**	84	05
		- 14 UD	- 02 UD
		- 05 non-AARD	- 03 non-AARD
		- 65 AARD	
	**(–)**	73	109
		- 15 UD	- 02 UD
		- 29 non-AARD	- 107 non-AARD
		- 29 AARD	
UNI	**(+)**	89	04
		- 11 UD	- 01 UD
		- 07 non-AARD	- 03 non-AARD
		- 71 AARD	
	**(–)**	68	110
		- 18 UD	- 03 UD
		- 27 non-AARD	- 107 non-AARD
		- 23 AARD	

AARD, antinuclear antibody-associated rheumatic disease; ANA, antinuclear antibodies; UD, undetermined.

As shown in **Table 4**, the positivity of CLIA MAGLUMI in patients with confirmed AARD (Group 1) was the highest (95.7%), close to the IIF positivity rate (100%) followed by the automated EIA UNI and CLIA iFlash with a positivity rate of 75.5% and 69.1% respectively. Among patients in whom the diagnosis of AARD was excluded (Group 2), the positivity rates were 21.5%, 6.9%, 5.6% for the CLIA MAGLUMI, EIA UNI and CLIA iFlash, respectively, *versus* 23.6% with the IIF assay. In the group of patients in whom the diagnosis remained unestablished (Group 3), the positivity of CLIA MAGLUMI, CLIA iFlash and EIA UNI was 78.8%, 48.5% and 36.4%, respectively, *versus* 87.9% of positivity with the IIF assay.

**Table 4:** Overview of positive rates in each disease group for ANA HEp-2 (IIF), MAGLUMI ANA screen (CLIA), iFlash ANA screen (CLIA) and UNI ANA HEp-2 (EIA).

Disease	N	HEp-2		MAGLUMI		iFlash		UNI	
		n Pos	% Pos	n Pos	% Pos	n Pos	% Pos	n Pos	% Pos
AARD	94	94	100%	90	95.70%	65	69.10%	71	75.50%
SLE	31	31	100%	28	90.30%	23	74.20%	20	64.50%
SS	29	29	100%	29	100%	27	93.10%	26	89.70%
SSc	19	19	100%	19	100%	4	21.10%	16	84.20%
MCTD	8	8	100%	8	100%	7	87.50%	6	75%
PM (AS)	2	2	100%	1	50%	0	0%	0	0%
Two diseases	5	5	100%	5	100%	4	80%	3	60%
Non-AARD	144	34	23.60%	31	21.50%	8	5.60%	10	6.90%
Undetermined	33	29	87.90%	26	78.80%	16	48.50%	12	36.40%

AARD, antinuclear antibody-associated rheumatic disease; ANA, antinuclear antibodies; AS, antisynthetase syndrome; CLIA, Chemiluminescence immune-assay; IIF, indirect immunofluorescence; MCTD, mixed connective tissue disease; PM, polymyositis; SS, primary Sjogren’s syndrome; SLE, systemic lupus erythematosus; SSc, systemic sclerosis.

### 3.2 Concordance and agreement

The MAGLUMI ANA assay showed the highest positivity rate in both AARD and non AARD patients. Concerning the concordance and agreement with the IIF assay (**Table 5**), the three automated ANA assays showed a weak agreement with Cohen’s Kappa ranging from 0.4 to 0.59: (ĸ =0.551; 95% CI 0.451-0.651]) with the MAGLUMI ANA assay, (ĸ =0.494; 95% CI [0.404-0.584]) with the UNI assay and (ĸ =0.454; 95% CI [0.354-0.554]) with the iFlash assay. It should be noted that the MAGLUMI ANA assay showed the highest agreement with the IIF when using the manufacturer’s cutoff values.

**Table 5:** Qualitative agreement between methods.

Method	HEp-2	MAGLUMI	iFlash
UNI			
Total concordance rate; n (%) • Agreement	• 199 (73.4%) • ĸ =0.494 *p*<0.001 95% CI [0.404-0.584]	• 197 (72.7%) • ĸ =0.468 *p*<0.001 95% CI [0.372-0.564]	• 227 (83.8%) • ĸ =0.636 *p*<0.001 95% CI [0.538-0.734]
iFlash			
Total concordance rate; n (%) • Agreement	• 193 (71.2%) • ĸ =0.454 *p*<0.001 95% CI [0.354-0.554]	• 199 (73.4%) • ĸ =0.484 *p*<0.001 95% CI [0.384-0.584]	
MAGLUMI			
Total concordance rate; n (%) • Agreement	• 211 (77.9%) • ĸ =0.551 *p*<0.001 95% CI [0.451-0.651]		

### 3.3 Diagnostic performance characteristics of assays

To compare the diagnostic performance of the tests, ROC analysis was performed (**Table 6**). All ANA assays showed excellent performance in discriminating AARD from non-AARD (AUC>0.9 for each system, ranging from 0.905 to 0.967): 0.967 [95% CI (0.948-0.987)] for the MAGLUMI assay, followed by 0.927 [95% CI (0.888-0.967)] for the UNI assay and followed by the iFlash assay 0.905 [95% CI (0.864-0.946)].

**Table 6:** Diagnostic performance characteristics.

Method	AUC (95% CI)	Cut-off value	Sensitivity	Specificity	LR+	LR-
MAGLUMI X3	0.967 (0.948-0.987)	≥ 40 AU/mL (manufacturer)	95.70%	78.50%	4.451	0.055
		≥ 227.5 AU/mL (optimal)	83%	97.90%	39.523	0.173
iFlash	0.905 (0.864-0.946)	> 48 AU/mL (manufacturer)	69.10%	94.40%	12.339	0.327
		≥ 30.9 AU/mL (optimal)	75.50%	93.10%	10.942	0.263
UNI	0.927 (0.888-0.967)	> 18 U/mL (manufacturer)	75.50%	93.10%	10.942	0.263
		≥ 6.2 U/mL (optimal)	90.40%	87.50%	7.232	0.11

At the manufacturer’s cut-off values, the MAGLUMI assay showed the highest sensitivity (95.7%) followed by the UNI assay (75.5%) and the iFlash assay with a sensitivity of (69.1%). The specificities were 94.4% for the iFlash, 93.1% for the UNI and 78.5% for the MAGLUMI assay. Only The MAGLUMI assay showed a low negative likelihood ratio (<0.1) with a positive likelihood ratio of 4.451, whereas the UNI and the iFlash ANA assays showed a high positive likelihood ratio (≥10).

**Figure 3:**
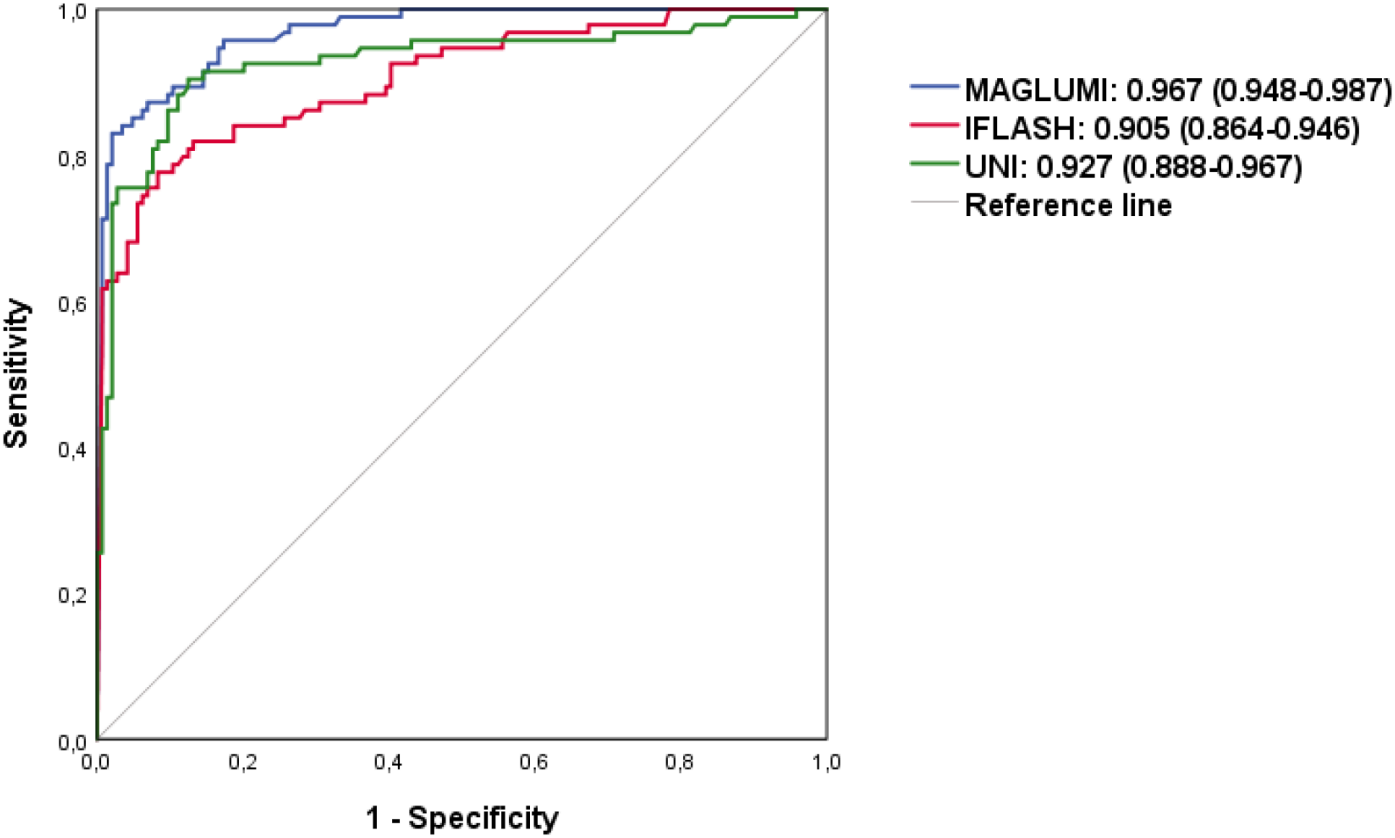
ROC curves analysis.

Using the ROC curve analysis (**Figure 3**), we determined optimal cut-offs for each test allowing a variation of sensitivity, the highest with the UNI assay (90.4%) at a cut-off of 6.2 U/mL, followed by the MAGLUMI assay with a sensitivity of 83% at 227.5 AU/mL, followed by iFlash (75.5%) at a cut-off value of 30.9 AU/mL. The specificities were 97.9% for the MAGLUMI assay, 93.1% for the iFlash assay and 87.5% for the UNI assay.

### 3.4 Analysis of discrepant results

In total, we found discrepant results between the four assays in 121 samples: 60 samples with discrepant MAGLUMI and IIF results, 72 samples with discrepant UNI and IIF results and 78 samples with discrepant iFlash and IIF results. The results, the retained/definitive diagnosis and the ANA’s targets identified are listed in **Table 7**. The results are grouped according to the different combinations of positive/negative results on the different platforms (IIF and SPA). In each group, we specified the proportion of AARD and non-AARD patients, as well as the antigenic targets found.

**Table 7:** Summary table of discrepant results between ANA assays.

Profil	N	IIF	MAGLUMI	iFlash	UNI	DIAGNOSIS			IDENTIFICATION								
						AARD	Non-AARD	UD	Negative	Positive*							
										dsDNA	SSA	SSB	Sm	Sm/RNP	Scl70	CENP	Jo-1
1	24	P	N	N	N	3	16	5	19	3	1	0	0	0	0	0	1
2	22	N	P	N	N	0	20	2	15	1	0	0	0	2	7	0	0
3	1	N	N	P	N	0	1	0	1	0	0	0	0	0	0	0	0
4	1	N	N	N	P	0	1	0	1	0	0	0	0	0	0	0	0
5	26	P	P	N	N	12	7	7	12	7	3	1	1	2	7	0	1
6	3	P	N	P	N	1	1	1	1	2	0	0	0	0	0	0	0
7	6	P	N	N	P	0	6	0	5	0	0	0	0	1	0	0	0
8	1	N	P	P	N	0	0	1	0	0	0	0	0	1	0	0	0
9	0	N	P	N	P	/	/	/	/	/	/	/	/	/	/	/	/
10	1	N	N	P	P	0	1	0	1	0	0	0	0	0	0	0	0
11	15	P	P	P	N	7	3	5	4	2	7	0	1	4	2	0	0
12	17	P	P	N	P	14	0	3	1	1	1	1	0	1	8	5	0
13	2	P	N	P	P	0	1	1	2	0	0	0	0	0	0	0	0
14	2	N	P	P	P	0	1	1	0	1	1	0	0	1	0	0	0

*Some patients tested positive for multiple antigens.

AARD, antinuclear antibody-associated rheumatic disease; N, negative; P, positive; UD, undetermined.

35 samples tested negative on MAGLUMI with positive IIF, with 8 (22.8%) tested positive for anti-DNA and/or anti-ENA assays with specificity for dsDNA (n=5), SSA (n=1), Sm/RNP (n=1) and Jo-1 (n=1) antigens. Only four patients (11.4%) had an established diagnosis of AARD.

Among the 73 samples tested negative on iFlash with positive IIF, 36 (49.3%) samples were positive for anti-DNA and/or anti-ENA assays with specificity for Scl70 (n=15), dsDNA (n=11), centromeres (n=5), SSA (n=5), Sm-RNP (n=4), SSB (n=2), Jo-1 (n=2), and Sm (n=1) antigens. 29 patients (39.7%) had an established diagnosis of AARD.

Concerning the UNI assay, 68 samples tested negative on UNI with positive IIF, 32 (47%) samples were positive for anti-DNA and/or anti-ENA assays with specificity for dsDNA (n=14), SSA (n=11), Scl70 (n=9), Sm-RNP (n=6), Sm (n=2), Jo-1 (n=2) and SSB (n=1) antigens. In this group, 23 patients (33.8%) had an established diagnosis of AARD.

Besides, 28 samples tested negative on IIF but positive in at least one of the other systems. Among them, 10 (35.7%) patients were positive for anti-DNA and/or anti-ENA assays. It is interesting to specify that none of these patients had a retained diagnosis of AARD.

## 4 Discussion

To this day, the screening for antinuclear antibodies in connective tissue diseases by detection via indirect immunofluorescence assay using Human Epithelial cells type 2 has been the only one method recognized as the gold standard (9). However, the reported drawbacks of this assay, notably its subjectivity of interpretation, the fact that it is time-consuming and its low specificity, have prompted researchers to develop automated assays as alternative for the screening of ANA (6). Before implementing these assays in diagnostic algorithms of AARD, it is necessary to perform procedures of comparison with reference assays, validation and standardization.

Several studies have attempted to compare solid phase assays to IIF for the detection of antinuclear antibodies (2, 10–13). The study conducted by Op De Beeck et al. in 2011 reported that automated immunoassays are less sensitive than IIF for detecting ANA. However, in our study, the sensitivity of the CLIA MAGLUMI assay was found to be almost equal to that of IIF in each disease group (AARD, non-AARD, undetermined diagnosis).

Indeed, when analyzing the positivity rates in the AARD group, the CLIA MAGLUMI assay showed the highest percentage of positivity, reaching 96% and being the closest to the IIF assay (100%), making the MAGLUMI a reliable assay for ANA detection. The CLIA iFlash and UNI automated EIA assays showed a lack of sensitivity with a positivity rate of 70-75% in the AARD group.

When analyzing the percentage of positivity according to each connective tissue disease in the AARD group, we noted that the percentage of positivity was lower in SLE compared to other connective tissues diseases. (90.3% with the MAGLUMI assay, 74.2% with the iFlash assay and 64.5% with the UNI assay *versus* 100% with the IIF assay). This is consistent with the literature data (10, 14). This can be explained by the kinetics of auto-antibodies in the course of SLE, notably, in response to treatment.

An exception was observed for the iFlash assay where the positivity rate was of 21% in systemic sclerosis (**Table 4**), which suggests a poor antigenic representation of antigens associated with systemic sclerosis, namely Scl70 and centromeres (15). It should be noted that the iFlash assay contains only centromere B antigens, while the other assays contain all the centromeric antigens.

Anti-centromere antibodies associated with systemic sclerosis are mainly directed against three proteins (CENP-A, CENP-B, and CENP-C), among which CENP-B is considered the major epitope (16).

Regarding the anti-synthetase syndrome and the association of two AARD, the number of patients was not representative enough to draw conclusions (n=2, n=5 respectively).

On the contrary, the study conducted by Yoon et al. in 2022, which focused on the comparison of three assays: EIA, CLIA and IIF, found a lower rate of ANA positivity with the CLIA assay across the different groups of patients compared to the IIF assay. In our study, the CLIA MAGLUMI assay reached the positivity rate of the IIF assay in the non-AARD group (=20%), whereas the CLIA iFlash assay and the automated EIA UNI assay had a significantly lower positivity rate (=6%) in this same group. The iFlash and UNI assays proved to be superior in eliminating false positives, whereas with the CLIA MAGLUMI, the specificity problem described for the IIF assay persisted.

In our study, by jointly analyzing the clinical data as well as the results of the identification assays in the face of discrepant results between the three solid phase assays tested and the IIF assay. We observed, on the one hand, that the screening assays evaluated did not provide more information than the reference IIF ANA assay in the diagnosis of connective tissue diseases. In fact, IIF was positive in 100% of patients with connective tissue diseases, unlike studies where solid phase assays allowed the diagnosis of a small number of patients with AARD who had an IIF ANA screening test negative (2, 17, 18).

On the other hand, we were able to highlight the interest of these assays in improving the specificity of the ANA IIF assay: among 44 patients who tested positive by IIF, but for whom the identification panel was negative and the diagnosis of connective tissue disease was not retained, 84% (37/44) were negative on iFlash, 81.8% (36/44) were negative on UNI and 61.4% (27/44) negative on MAGLUMI.

This improvement in specificity appears to be associated with the fact that SPA tests are enriched with antigens specific to connective tissue diseases, thus reflecting the added value of SPA tests.

However, SPA tests missed true positive patients with an established diagnosis of AARD. This percentage was 4.3% for the MAGLUMI test and 6 to 7 times higher for the UNI (24.5%) and the iFlash (30.9%) tests. We clearly observed the highest specificity with the iFlash and UNI assays, but in some cases this came at the expense of sensitivity. Adjusting cut-off thresholds could lead to a better compromise between sensitivity and specificity.

Regarding the MAGLUMI assay, its higher sensitivity may be due to the use of an antigenic substrate enriched with HEp-2 nuclear extracts associated with the CLIA principle. Indeed, it has been reported that SPA like ELISAs that incorporate cellular extracts of HEp­2 or HeLA cells, have a high sensitivity (90%) but a low specificity, whereas an ELISA that incorporates only a mixture of separate antigens shows a lower sensitivity (76%) and higher specificity (90.4%) than HEp­2 IIF. Thus, it is important to know the type of antigens coated on the solid phase used for ANA screening to adequately interpret the test results (7, 12).

The UNI test also incorporates HEp-2 cell extracts, but the results of our study did not reveal an improvement in its sensitivity. This can be explained by several parameters, including the type of substrates used and the manufacturing process, such as the purification of native antigens and post-synthetic modifications for recombinant antigens. Moreover, there seems to be a different affinity of the antigens depending on the solid phase used. Finally, the principle of the technique, which in this case is EIA, differs from the MAGLUMI test, which uses chemiluminescence (7, 10).

Ten patients tested negative by IIF and positive on the identification panel. The targets found were Scl70 in 70% (7/10), Sm/RNP in 40% (4/10), dsDNA in 20% (2/10) and SSA in 10% (1/10). Among the 10 patients, 6 were patients for whom the diagnosis of AARD was excluded and 4 were classified as having an undetermined diagnosis at the time of the study. It should be noted that 50% (2/4) of these patients were positive in another solid phase system: one in iFlash and MAGLUMI and one in the three SPA systems. These results raise the idea that there is a need for a follow-up of these patients who are positive for CLIA screening and negative by IIF assay. Indeed, Bizzaro et al., 2018 found that five patients who were ANA-IIF negative (but CTD screen positive) at enrollment were diagnosed with AARD during follow up.

These results, together with the experience acquired using solid-phase screening tests, lead us to believe that a complementary strategy between the IIF and solid-phase assays, particularly CLIA on the MAGLUMI platform, could be valuable for ANA screening in AARD. Performing the automated assay first would save time and help exclude negative patients. Subsequently, the IIF-HEp 2 could be employed in cases of discrepancy with the clinical presentation, with the identification panel and also in cases where the antigen is not represented in the screening assay, particularly some nucleolar antigens associated with systemic sclerosis, antigens linked to inflammatory myopathies and the case of the DFS70 Antigen (19–22). In these situations, the specific immunofluorescence patterns, together with the clinical context, can guide targeted antigen identification. A strategy based on the clinical suspicion, given the multitude of IIF patterns and the antigenic targets associated with each AARD, combined with knowledge of the clinical performance of each assay, is therefore recommended (23). Besides, in some cases of AARD, notably in systemic sclerosis, primary Sjogren’s syndrome and the IPAF entity (Interstitial Pneumonia with Autoimmune Features), the only autoimmune feature detected may be the positivity of ANA at a significant titer without any identified target (24). Such a strategy would have the advantage of avoiding false negatives on the IIF test, particularly with some highly soluble or poorly represented antigens, such as the SSA and Jo-1 targets, provided that these antigens are well represented in the SPA test (2, 6, 8, 25).

In our study, the IIF test allowed the diagnosis of 100% of patients with AARD. However, it is important to note that the effective population of patients tested was small (29 patients with SS and 2 patients with anti-synthetase syndrome) and that the definitive diagnosis was considered when classifying patients.

An algorithm combining the IIF assay with the SPA assays has already been proposed in other studies (1–3, 10) which even provided proof of an improvement in the cost/benefit ratio for the diagnosis of AARD (26), concluding that the SPA can be advantageously associated with IIF when the ANA request has a low pre-test probability and when the requests do not report useful clinical information for a targeted disease-oriented search for antibody specificity, a diagnosis strategy that we want to adopt in our laboratory, using the CLIA MAGLUMI assay, which showed the highest sensitivity and it is well adapted to a screening assay (8).

**Figure 4:**
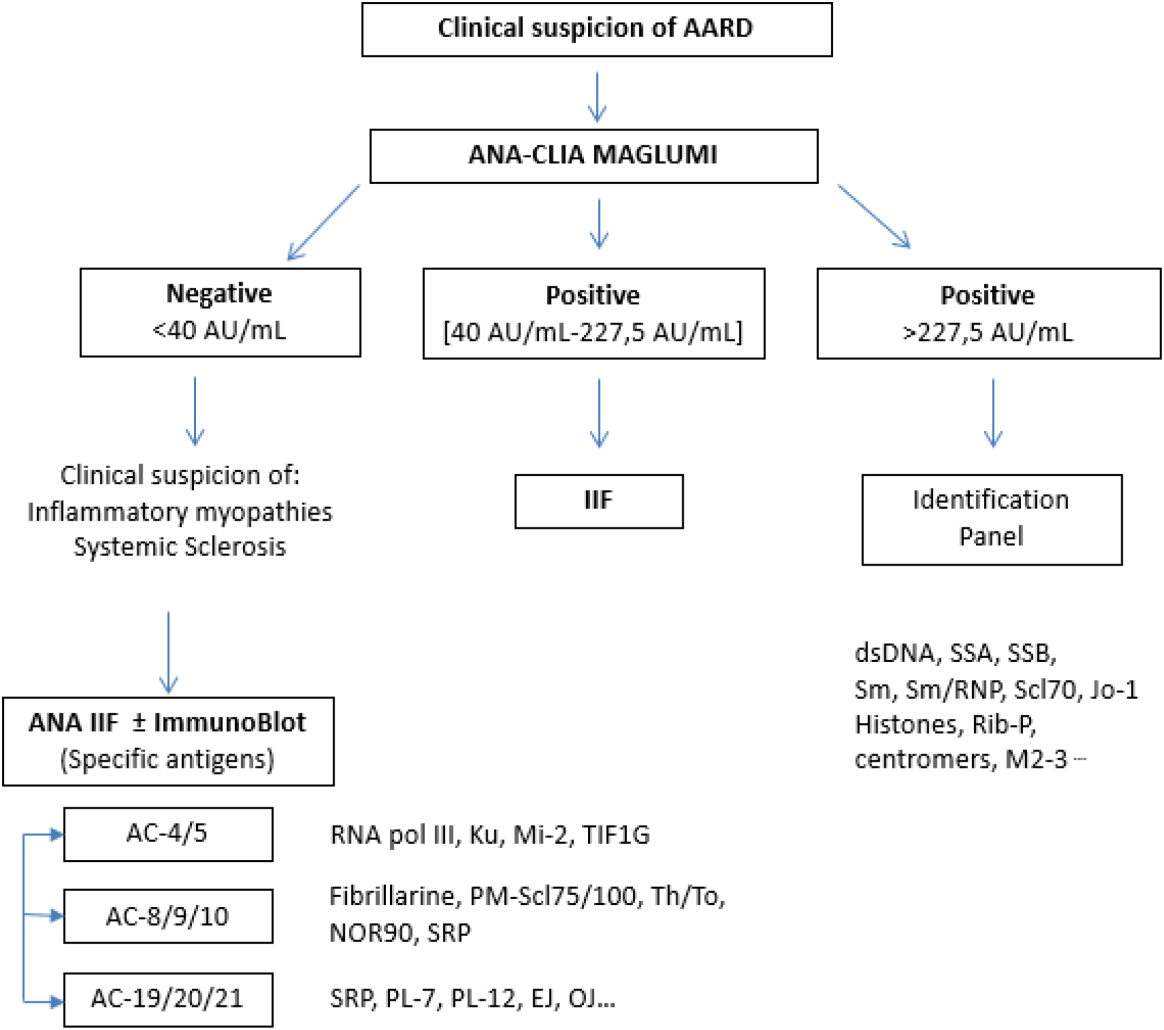
Proposed algorithm for ANA screening using MAGLUMI assay.

We propose an algorithm (**Figure 4**) combining the MAGLUMI and IIF assays, using the optimal cut-off values calculated and an IIF pattern-oriented strategy, which allows the identification of a higher number of antibodies not detected by ENA testing. This approach is a reliable and modern tool for managing ANA positivities according to the international ICAP classification (27). Concerning iFlash and UNI assays, we suggest an improvement of the antigenic component.

This study has some limitations, notably the sample size, in particular, the number of patients with AARD. A multicenter study on a larger cohort with a representative sample for each AARD is necessary in order to evaluate the clinical performance of these assays in each individual AARD. Furthermore, a short to medium-term follow up of patients tested positive is recommended, giving the predictive value of antibodies in AARD. Finally, the analytical performance of these automated assays should be assessed.

## 5 Conclusion

This comparative study highlights the added value of jointly screening for ANA by IIF and CLIA MAGLUMI, given the high sensitivity of these assays. UNI and iFlash assays showed excellent specificity, but improvement in their antigen composition is recommended to increase their sensitivity.

A complementary strategy between the IIF and SPA techniques, given the automation of SPA, can provide objectivity in the ANA screening tests and enable the diagnosis of the maximum number of patients in a short period of time.

However, it is still too early to conclude that SPA represent a reliable alternative to the IIF assay. The IIF assay remains essential in many indications where SPA tests remain limited.

## Data Availability

The original contributions presented in the study are included in the article/supplementary material. Further inquiries can be directed to the corresponding author/s.

## References

[1] Bonroy C Vercammen M Fierz W Andrade LEC Van Hoovels L Infantino M. Detection of antinuclear antibodies: recommendations from EFLM, EASI and ICAP. Clin Chem Lab Med. (2023) 61:1167−98. doi: 10.1515/cclm-2023-0209, PMID: 36989417

[2] Bizzaro N Brusca I Previtali G Alessio MG Daves M Platzgummer S. The association of solid-phase assays to immunofluorescence increases the diagnostic accuracy for ANA screening in patients with autoimmune rheumatic diseases. Autoimmun Rev. (2018) 17:541−7. doi: 10.1016/j.autrev.2017.12.007, PMID: 29631063

[3] Damoiseaux J Andrade LEC Carballo OG Conrad K Francescantonio PLC Fritzler MJ. Clinical relevance of HEp-2 indirect immunofluorescent patterns: the International Consensus on ANA patterns (ICAP) perspective. Ann Rheum Dis. (2019) 78:879−89. doi: 10.1136/annrheumdis-2018-214436, PMID: 30862649 PMC6585284

[4] Oyaert M Bossuyt X Ravelingien I Van Hoovels L. Added value of indirect immunofluorescence intensity of automated antinuclear antibody testing in a secondary hospital setting. Clin Chem Lab Med févr. (2016) 54:e63–66. doi: 10.1515/cclm-2015-0887, PMID: 26562037

[5] Krause C Ens K Fechner K Voigt J Fraune J Rohwäder E. EUROPattern Suite technology for computer-aided immunofluorescence microscopy in autoantibody diagnostics. Lupus. (2015) 24:516−29. doi: 10.1177/0961203314559635, PMID: 25801895

[6] Bossuyt X. Understanding and interpreting antinuclear antibody tests in systemic rheumatic diseases. Nat Rev. (2020) 16:715–26. doi: 10.1038/s41584-020-00522-w, PMID: 33154583

[7] Bernardini S Infantino M Bellincampi L Nuccetelli M Afeltra A Lori R. Screening of antinuclear antibodies: comparison between enzyme immunoassay based on nuclear homogenates, purified or recombinant antigens and immunofluorescence assay. Clin Chem Lab Med. (2004) 42:1155−60. doi: 10.1515/CCLM.2004.235, PMID: 15552275

[8] Tonuttia E Bassetti D Piazza A Visentini D Poletto M Bassetto F. Diagnostic accuracy of ELISA methods as an alternative screening test to indirect immunofluorescence for the detection of antinuclear antibodies. Evaluation of five commercial kits. Autoimmunity. (2004) 37:171−6. doi: 10.1080/08916930310001657010, PMID: 15293886

[9] Meroni PL Schur PH. ANA screening: an old test with new recommendations. Ann Rheum Dis. (2010) 69:1420−2. doi: 10.1136/ard.2009.127100, PMID: 20511607

[10] Yoon S Moon HW Kim H Hur M Yun YM. Clinical performance of two automated immunoassays, eliA CTD screen and QUANTA flash CTD screen plus, for antinuclear antibody screening. Ann Lab Med. (2022) 42:63−70. doi: 10.3343/alm.2022.42.1.63, PMID: 34374350 PMC8368234

[11] Claessens J Belmondo T De Langhe E Westhovens R Poesen K Hüe S. Solid phase assays versus automated indirect immunofluorescence for detection of antinuclear antibodies. Autoimmun Rev. (2018) 17:533−40. doi: 10.1016/j.autrev.2018.03.002, PMID: 29526634

[12] de Almeida Brito F Maria Elói Santos S Aparecida Ferreira G Pedrosa W Gradisse J Cristina Costa L. Diagnostic evaluation of ELISA and chemiluminescent assays as alternative screening tests to indirect immunofluorescence for the detection of antibodies to cellular antigens. Am J Clin Pathol. (2016) 145:323−31. doi: 10.1093/ajcp/aqv083, PMID: 27124914

[13] Willems P De Langhe E Claessens J Westhovens R Van Hoeyveld E Poesen K. Screening for connective tissue disease-associated antibodies by automated immunoassay. Clin Chem Lab Med. (2018) 56:909−18. doi: 10.1515/cclm-2017-0905, PMID: 29306915

[14] González C Garcia-Berrocal B Herráez O Navajo JA González-Buitrago JM. Anti-nucleosome, anti-chromatin, anti-dsDNA and anti-histone antibody reactivity in systemic lupus erythematosus. Clin Chem Lab Med. (2004) 42:266−72. doi: 10.1515/CCLM.2004.049, PMID: 15080558

[15] van den Hoogen F Khanna D Fransen J Johnson SR Baron M Tyndall A. 2013 classification criteria for systemic sclerosis: an American college of rheumatology/European league against rheumatism collaborative initiative. Ann Rheum Dis. (2013) 72:1747−55. doi: 10.1136/annrheumdis-2013-204424, PMID: 24092682

[16] Cavazzana I Vojinovic T Airo’ P Fredi M Ceribelli A Pedretti E. Systemic sclerosis-specific antibodies: novel and classical biomarkers. Clin Rev Allergy Immunol. (2023) 64:412−30. doi: 10.1007/s12016-022-08946-w, PMID: 35716254 PMC10167150

[17] Op De Beeck K Vermeersch P Verschueren P Westhovens R Mariën G Blockmans D. Detection of antinuclear antibodies by indirect immunofluorescence and by solid phase assay. Autoimmun Rev. (2011) 10:801−8. doi: 10.1016/j.autrev.2011.06.005, PMID: 21741497

[18] Bossuyt X Fieuws S. Detection of antinuclear antibodies: added value of solid phase assay? Ann Rheum Dis. mars. (2014) 73:e10. doi: 10.1136/annrheumdis-2013-204793, PMID: 24257025

[19] Bodakçi E. Clinical and serological characteristics of anti-Ro/SS-A and anti-La/SS-B negative primary Sjögren’s syndrome: a comparative study. Eur Rev Med Pharmacol Sci. (2024) 28:1760−7. doi: 10.26355/eurrev_202403_35589, PMID: 38497858

[20] Cheng CF Shih MC Lan TY Li KJ. Anti-DFS70 antibodies for differentiating systemic autoimmune rheumatic disease in patients with positive ANA tests: A systematic review and meta-analysis. Diagnostics (Basel). (2021) 11:1592. doi: 10.3390/diagnostics11091592, PMID: 34573934 PMC8468616

[21] Bossuyt X. DFS70 autoantibodies: clinical utility in antinuclear antibody testing. Clin Chem. (2024) 70:374−81. doi: 10.1093/clinchem/hvad181, PMID: 38084885

[22] Infantino M Tampoia M Fabris M Alessio MG Previtali G Pesce G. Combining immunofluorescence with immunoblot assay improves the specificity of autoantibody testing for myositis. Rheumatol (Oxford). (2019) 58:1239−44. doi: 10.1093/rheumatology/key451, PMID: 30726990

[23] Robier C Amouzadeh-Ghadikolai O Stettin M Reicht G. Comparison of the clinical utility in the detection of anti-nuclear antibodies between the elia CTD screen and indirect immunofluorescence on hep-2 cells: A review of the literature. Isr Med Assoc J. (2018) 20:700−2. doi: 10.1515/cclm-2015-1051, PMID: 30430800

[24] Jeganathan N Sathananthan M. Connective tissue disease-related interstitial lung disease: prevalence, patterns, predictors, prognosis, and treatment. Lung. (2020) 198:735−59. doi: 10.1007/s00408-020-00383-w, PMID: 32780179

[25] Agmon-Levin N Damoiseaux J Kallenberg C Sack U Witte T Herold M. International recommendations for the assessment of autoantibodies to cellular antigens referred to as anti-nuclear antibodies. . Ann Rheum Dis. (2014) 73:17−23. doi: 10.1136/annrheumdis-2013-203863, PMID: 24126457

[26] Gallo N Musso G. Plebani M. A cost-effective assessment for the combination of indirect immunofluorescence and solid-phase assay in ANA-screening. Clin Chem Lab Med. (2025) 63:1974–80. doi: 10.1515/cclm-2025-0170, PMID: 40418776

[27] Infantino M Carbone T Manfredi M Grossi V Antico A Panozzo MP. A new diagnostic algorithm for pattern-oriented autoantibody testing according to the ICAP nomenclature: A pilot study. Autoimmun Rev août. (2020) 19:102588. doi: 10.1016/j.autrev.2020.102588, PMID: 32540447

